# Intelligibility of speech produced by sighted and blind adults

**DOI:** 10.1371/journal.pone.0272127

**Published:** 2022-09-15

**Authors:** Lucie Ménard, Pamela Trudeau-Fisette, Mark Kenneth Tiede

**Affiliations:** 1 Laboratoire de Phonétique, Université du Québec à Montréal, Center for Research on Brain, Language, and Music, Montreal, Canada; 2 Haskins Laboratories, New Haven, CT, United States of America; Singapore Institute of Technology, SINGAPORE

## Abstract

**Purpose:**

It is well known that speech uses both the auditory and visual modalities to convey information. In cases of congenital sensory deprivation, the feedback language learners have access to for mapping visible and invisible orofacial articulation is impoverished. Although the effects of blindness on the movements of the lips, jaw, and tongue have been documented in francophone adults, not much is known about their consequences for speech intelligibility. The objective of this study is to investigate the effects of congenital visual deprivation on vowel intelligibility in adult speakers of Canadian French.

**Method:**

Twenty adult listeners performed two perceptual identification tasks in which vowels produced by congenitally blind adults and sighted adults were used as stimuli. The vowels were presented in the auditory, visual, and audiovisual modalities (experiment 1) and at different signal-to-noise ratios in the audiovisual modality (experiment 2). Correct identification scores were calculated. Sequential information analyses were also conducted to assess the amount of information transmitted to the listeners along the three vowel features of height, place of articulation, and rounding.

**Results:**

The results showed that, although blind speakers did not differ from their sighted peers in the auditory modality, they had lower scores in the audiovisual and visual modalities. Some vowels produced by blind speakers are also less robust in noise than those produced by sighted speakers.

**Conclusion:**

Together, the results suggest that adult blind speakers have learned to adapt to their sensory loss so that they can successfully achieve intelligible vowel targets in non-noisy conditions but that they produce less intelligible speech in noisy conditions. Thus, the trade-off between visible (lips) and invisible (tongue) articulatory cues observed between vowels produced by blind and sighted speakers is not equivalent in terms of perceptual efficiency.

## 1. Introduction

In face-to-face conversation, speakers produce articulatory gestures that are heard and seen by the perceiver [1]. In quiet conditions, speech intelligibility scores are higher in audiovisual conditions than in auditory only conditions [2–5]. In noisy environments, the visual cues transmitted by lip and jaw movements, for instance, supplement the auditory cues [6–8]. However, speakers who exhibit atypical speech production, due to sensory deprivation or articulatory impairment, often have lower intelligibility scores. Individuals who have never had access to visual cues, for instance, have reduced (visible) lip gestures, but as a trade-off, they produce larger (invisible) tongue contrasts [9–12]. What are the consequences of such differences for the multimodal intelligibility of their speech?

### 1.1 Background

#### 1.1.1 Multimodal speech intelligibility

Speech intelligibility is multimodal. Perceivers use auditory as well as visual cues to process speech. Even though visual cues from the whole face (e.g., eyebrows and head movements; [13,14]) provide functional cues to speech perception, visual correlates of movements specifically related to the orofacial articulators are relevant in speech perception [15]. Even when video of only the lower face (jaw and lips) is presented, global correct speech identification scores increase in the audiovisual condition compared to the auditory condition [16]. Visual cues are also involved in another mechanism known as visual enhancement of auditory cues, whereby lip movements give the listener temporal cues that facilitate auditory detection of relevant acoustic events [6]. In a study of vowel perception in French under various noisy conditions, Robert-Ribès et al. (1998) [2] showed that auditory and visual speech cues are complementary: while some features (e.g., vowel height) are better perceived in the auditory modality, others (e.g., lip rounding) are better perceived in the visual modality. The authors’ experiments were carried out with French oral vowels that contrasted along three phonological dimensions: height, place of articulation and rounding (for the front non-low vowels only). Based on the results of identification tasks in three conditions (auditory alone, visual alone, and audiovisual) and in different noise levels, they proposed robustness scales for vowel features (the higher the correct identification score in noisy conditions, the greater the robustness). In the audio channel, height is the most robust feature, followed by place of articulation, which in turn is more robust than rounding. In the visual channel, rounding is the most robust feature, followed by height, while place of articulation is the least robust feature.

Those phonetic features (also referred to here as *dimensions*) are implemented by different positions of the orofacial articulators that have acoustic-auditory correlates as well as visual-optical correlates. In the acoustic channel, height contrasts are mainly related to variations of the first formant (F1). The place-of-articulation contrasts are mainly related to F2 [17], whereas the rounding dimension is related to both F2 and F3 [7]. Various linear combinations of formant frequencies, and even fundamental frequency (F0), have been shown to be perceptually correlated to those phonological features (as suggested, for instance, by [18–21]). In the visual domain, it has been shown that parameters such as the interolabial width (distance, in mm, between the right and left lip corners) and the interolabial height (distance, in mm, between the upper and lower lips) adequately describe lip geometry in French and English vowels [22–27]. Unrounded vowels are produced with larger interolabial width than their rounded counterparts, and interolabial height increases with vowel openness. Perceptual studies have confirmed the relevance of those parameters for the visual identification of rounding and openness [28–34].

#### 1.1.2 Speech production in congenitally blind speakers

In cases of congenital sensory deprivation, the movements of the orofacial articulators have been shown to be atypical and to affect the auditory and visual cues conveyed by vowels and consonants. For instance, studies provide evidence that congenital blindness affects the articulatory implementation of phonemes. In a first experiment [9], 12 congenitally blind adults (6 men; 6 women) and 12 sighted adult participants (6 men; 6 women) produced 10 repetitions of the 10 French oral vowels (/i y u e ø o ε œ ᴐ a/). At the acoustic level, the contrast distances produced, measured by the average vowel space (AVS, [35]) value, were significantly higher for sighted speakers than for blind speakers. Thus, vowels were spaced farther apart for sighted speakers than for blind ones. To further investigate lip-tongue relationships, the contributions of upper lip protrusion and tongue shape/position in the implementation of three French phonological vowel contrasts mainly involving those articulators (rounding, place of articulation, and rounding and place of articulation combined) were examined in a follow-up study [10]. First, it was found that the magnitude of the difference in upper lip protrusion (in mm) between the rounded and unrounded vowel pairs was significantly greater for sighted participants than for blind participants. Regarding place of articulation, tongue front-back position differences between those pairs were significantly greater for congenitally blind speakers than for their sighted peers. However, the contribution of upper lip protrusion was reduced for the blind speakers, suggesting a trade-off. Greater articulatory contrasts along the lip rounding dimension have also been found in a variety of contexts in French in sighted speakers compared to blind speakers: clear speech and conversational speech, fast speech, contrastive emphasis, etc. [10,11,36]. Similar effects of visual deprivation on vowel acoustics have also been found in Persian [37] and in Australian English [38]. However, the reverse was found in Dutch [39]: a larger acoustic vowel space in blind than in sighted adult speakers, thus suggesting that strategies to cope with visual deprivation are language-dependent.

The resulting effects of those group differences on intelligibility have not been directly addressed. Using previously known acoustic correlates of vowel perception in French, we analyzed vowels produced by sighted and blind adults in conversational and fast speech conditions [11]. Linear combinations of formant frequencies shown to be related to perceived height, place of articulation and rounding in French were used to classify the produced vowels. The results showed that, at increased speech rates, where intelligibility is jeopardized, blind speakers produced a larger proportion of vowels that were within the acoustic-auditory target regions of French. We interpreted those results as indicating that auditory templates were weighted more heavily in blind speakers’ speech representations than in those of their sighted peers. However, direct assessment of the intelligibility of speech produced by blind speakers, through identification scores for instance, has not been done.

#### 1.1.3 Objective and hypotheses

The findings presented thus far suggest that the lack of visual cues resulting from congenital blindness significantly influences the articulatory strategies speakers use to produce speech targets, especially in French. The question then arises: are the different strategies observed in blind and sighted speakers equally efficient perceptually? The objective of this paper is to address this question by determining the differences, in terms of intelligibility, between vowels produced by adult sighted and congenitally blind speakers of Canadian French. Based on the above-mentioned studies, our hypotheses are as follows:

1. Since the overall acoustic vowel space is larger in sighted adults than in blind adults, vowels produced by the former will be associated with higher intelligibility scores than those produced by the latter in the auditory modality;
2. Since vowels that contrast along the rounding feature are produced with larger lip contrasts by sighted speakers than by blind speakers, intelligibility scores in the visual modality associated with those vowels will be higher for sighted speakers than for blind speakers;
3. Since overall lip contrasts are smaller in blind speakers than in sighted speakers, intelligibility scores in the audiovisual modality when the audio signal is noisy (a condition in which listeners rely more heavily on visible lip movements) will be lower in blind speakers than in sighted speakers.

## 2. Method

### 2.1 Experiment 1

#### 2.1.1 Stimuli

The stimuli were a subset of vowels taken from the study described in Ménard et al. (2009) [9]. For the current experiments, the six French oral vowels /i e ε a y u/ were considered. Those vowels were chosen because they involve contrasts along three features: height, rounding, and place of articulation. Table 1 shows the articulatory descriptions of those vowels. The height dimension is represented by the contrast between /i/ vs. /e/ vs. /ε/ vs. /a/; the place of articulation dimension corresponds to the contrasts between /y/ vs. /u/; and the rounding dimension is represented by the /i/ vs. /y/ pair. Vowels were produced in the “*V* comme *mot*” [“*V* as in *word*”] context, where *V* corresponds to each of the six above-mentioned vowels, and *mot* is a word containing this vowel. For these experiments, only the vowels *V* were extracted and used as stimuli.

**Table 1:** Values for each phonetic feature of the six French oral vowels under study.

Vowel	Dimension		
	Height	Place of articulation	Rounding
/i/	High	Front	Unrounded
/e/	Mid-high	Front	Unrounded
/ε/	Mid-low	Front	Unrounded
/a/	Low	Front	Unrounded
/y/	High	Front	Rounded
/u/	High	Back	Rounded

Eight congenitally blind adults (6 men; 2 women), aged between 43 and 65, were recruited from our cohort of blind participants (see [9,10,12]). Because of technical issues, stimuli produced by only four sighted speakers from the 2009 study were included. We recorded four new sighted speakers to complete the data set. To avoid any technical bias, the exact same setup and material used in 2009 were also used for the current recordings. Blind speakers had a complete congenital visual impairment, classified as class 4 or 5 in the World Health Organization’s International Disease Classification. They had never had any perception of light or movement (see speakers’ characteristics in Table 2). All speakers lived in the Montreal area and were native speakers of Canadian French. They did not report any history of speech or language disorders. All participants were tested for pure-tone detection threshold using an adaptive method (DT < 25 dB HL at 250, 500, 1000, 2000, 4000, and 8000 Hz). Each speaker recorded 10 repetitions of the target sequence for each of the six vowels. A total of 960 stimuli (6 vowels x 10 repetitions x 16 speakers) were thus recorded. Each speaker’s lower face was recorded with a high-quality video camera, with the synchronized audio signal recorded using a high-quality microphone. The audio signal was digitized at a sampling frequency of 44100 Hz, and the video image was digitized at the standard NTSC sampling frequency (29.97 images per second). The speakers’ lips were painted in blue, following a method used in several audiovisual perception studies [40] and previously used to measure lip geometry in our studies [10]. The blue makeup allowed optimal detection of inner lip edges.

**Table 2:** Characteristics of the eight blind speakers.

Participant	Gender	Age	Etiology of blindness	Vision at birth	Current vision
S1B	F	48	retinitis pigmentosa	U	R.E. = 3/210 L.E. = 0
S2B	F	40	congenital cataract	U	R.E. = 0 L.E. = 6/1260
S3B	F	26	U	U	U (total blindness)
S4B	M	40	detachment of the retina	U	R.E. = 2/180 L.E. = 2/105
S5B	M	42	congenital cataract and congenital glaucoma	total blindness	U (total blindness)
S6B	F	51	retinitis pigmentosa	total blindness	R.E. = 2/400 L.E. = 2/400
S7B	F	45	congenital cataract	total blindness	U (total blindness)
S8B	F	45	congenital cataract	total blindness	R.E. = 0 L.E. = 0

L.E. = left eye; R.E. = right eye; U = undetermined.

**Fig 1:**
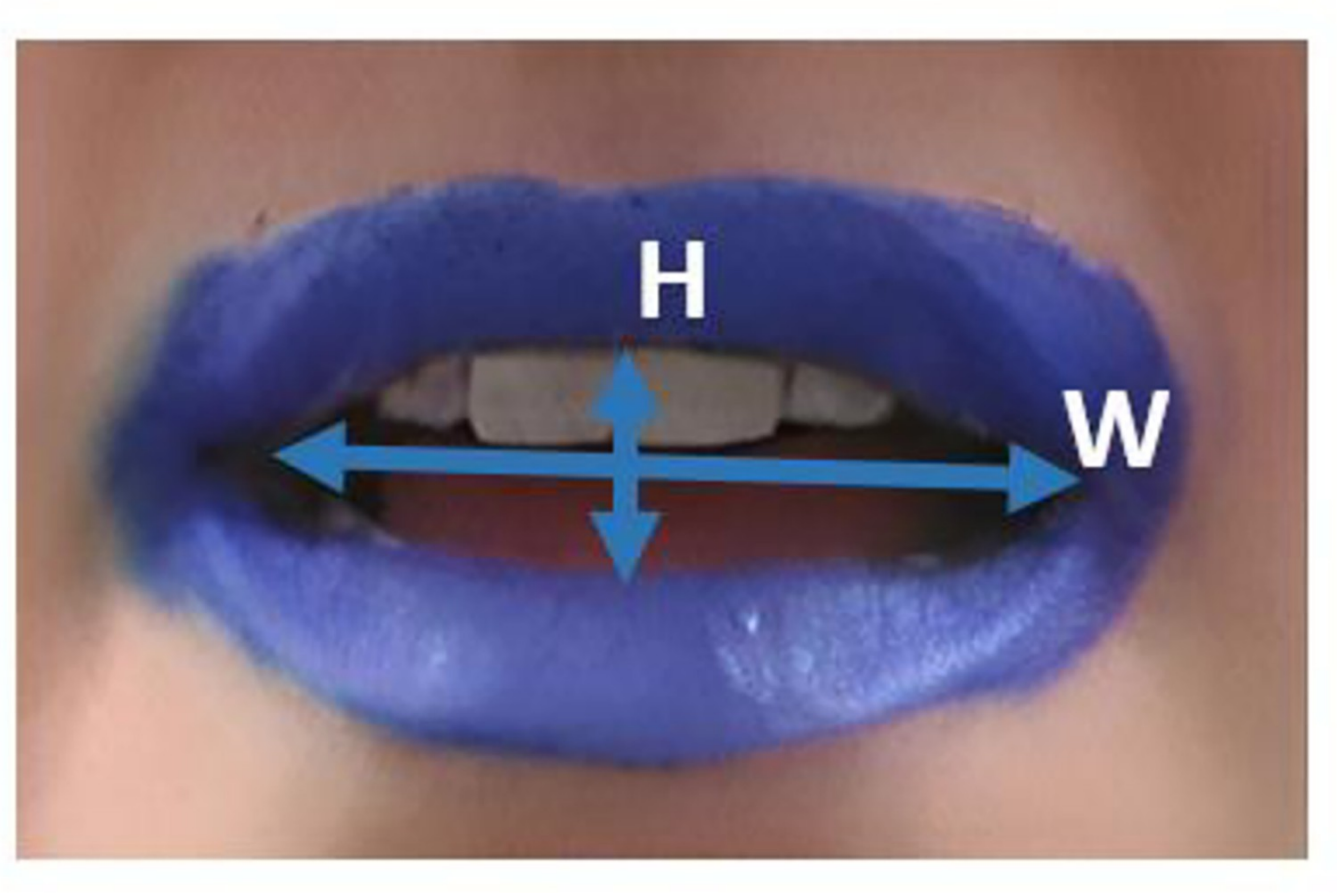
Articulatory measures extracted from the images of the lips: Interolabial height (H) and interolabial width (W).

In order to reduce the length of the perceptual experiment, three repetitions of each vowel produced by each speaker were selected: the fourth, fifth and sixth repetitions. Several measures were extracted on the audio and video signals to characterize the stimuli. As can be seen on the schematic representation provided in Fig 1, two measures were semi-automatically extracted from the image corresponding to vowel midpoint using custom-made Matlab programs: interolabial lip width, corresponding to the distance, in pixels, between the left and right mouth inner corners, and interolabial lip height, corresponding to the largest inner vertical distance between the upper lip and the lower lip.

**Fig 2:**
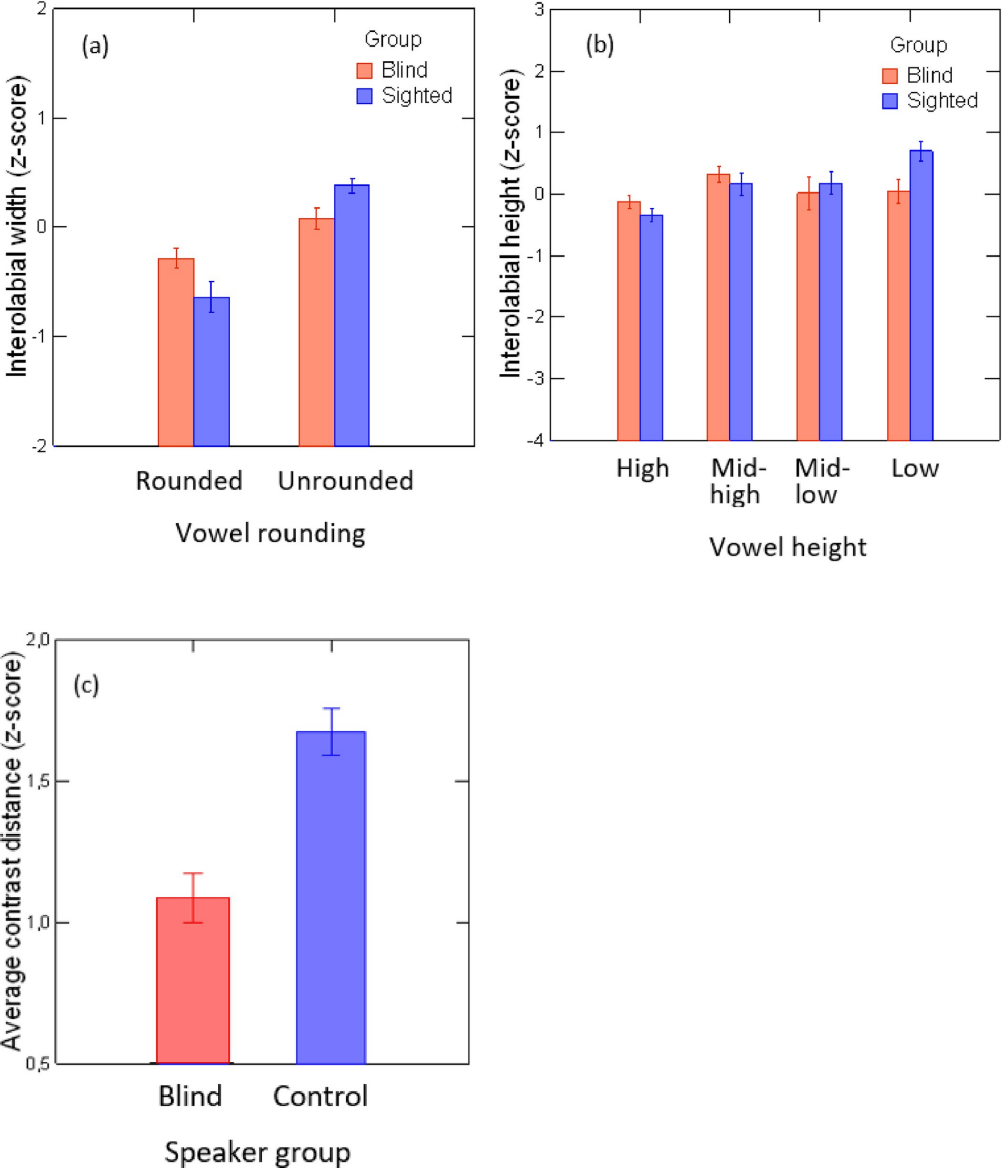
Articulatory and acoustic characteristics of vowel stimuli produced by blind and sighted speakers: Average values of interolabial width (a) and height (b) and average contrast distance (ACD) in the F1 vs. F2 space (c). All data were z-scored. Error bars are standard errors.

On the acoustical signal, the values of the first two formant frequencies (F1 and F2), in Hertz, were extracted using the Burg algorithm implemented in Praat [41]. All data were z-scored to normalize between-speaker variability. The size of the average contrast distance (ACD) between vowels was calculated by averaging, for each speaker, the Euclidean distance, in the F1 vs. F2 space, between all possible pairs of vowels among the six vowels under study. Average values for lip height, lip width and ACD are presented in Fig 2.

As can be seem from Fig 2A and 2B, lip width is significantly smaller for rounded vowels than unrounded vowels (χ^2^(1) = 36.33; p<0.001), and more so for sighted speakers than blind speakers (χ^2^(1) = 16.81; p<0.001). Similarly, vowel height has a significant effect on lip height in sighted speakers (with lip height increasing as vowels become lower), whereas no such effect is found in blind speakers (χ^2^(3) = 8.76; p<0.01). Concerning the acoustic contrast distance, as shown in Fig 2C, sighted speakers produce vowels that are spaced farther apart in the formant space than those produced by their blind peers (t = 5.21; p<0.001), which replicates the results presented in our previous studies (Ménard et al., 2009; 2014). The stimuli can thus be considered representative of vowels typically produced by sighted and congenitally blind adult speakers of Canadian French.

#### 2.1.2 Experimental procedure

A total of 20 participants (10 women; 10 men), aged between 21 and 33 years, took part in the perceptual identification task. All of them lived in the Montreal area and were native speakers of Canadian French. They did not report any history of speech or language disorders. All participants were tested for pure-tone detection threshold using an adaptive method (DT < 25 dB HL at 250, 500, 1000, 2000, 4000, and 8000 Hz). They all had perfect or near-perfect vision, or their vision was corrected using lenses or glasses.

The 960 vowels recorded by all speakers were randomly presented to listeners in three conditions: the auditory condition (audio signal alone), the visual condition (the video signal alone), and the audiovisual condition, using a PsychoPy interface [42]. Condition order was counterbalanced across participants. Their task was to identify from among a forced choice of seven responses (the six vowels /i e ε a y u/ and “other”) the vowel they heard, by selecting the corresponding button on the screen using a computer mouse. The experiment took place in the recording room at the Laboratoire de phonétique of Université du Québec à Montréal (UQAM) and lasted about 45 minutes, including breaks. The experiment was approved by UQAM’s institutional review board.

### Experiment 2

#### 2.2.1 Stimuli

**Fig 3:**
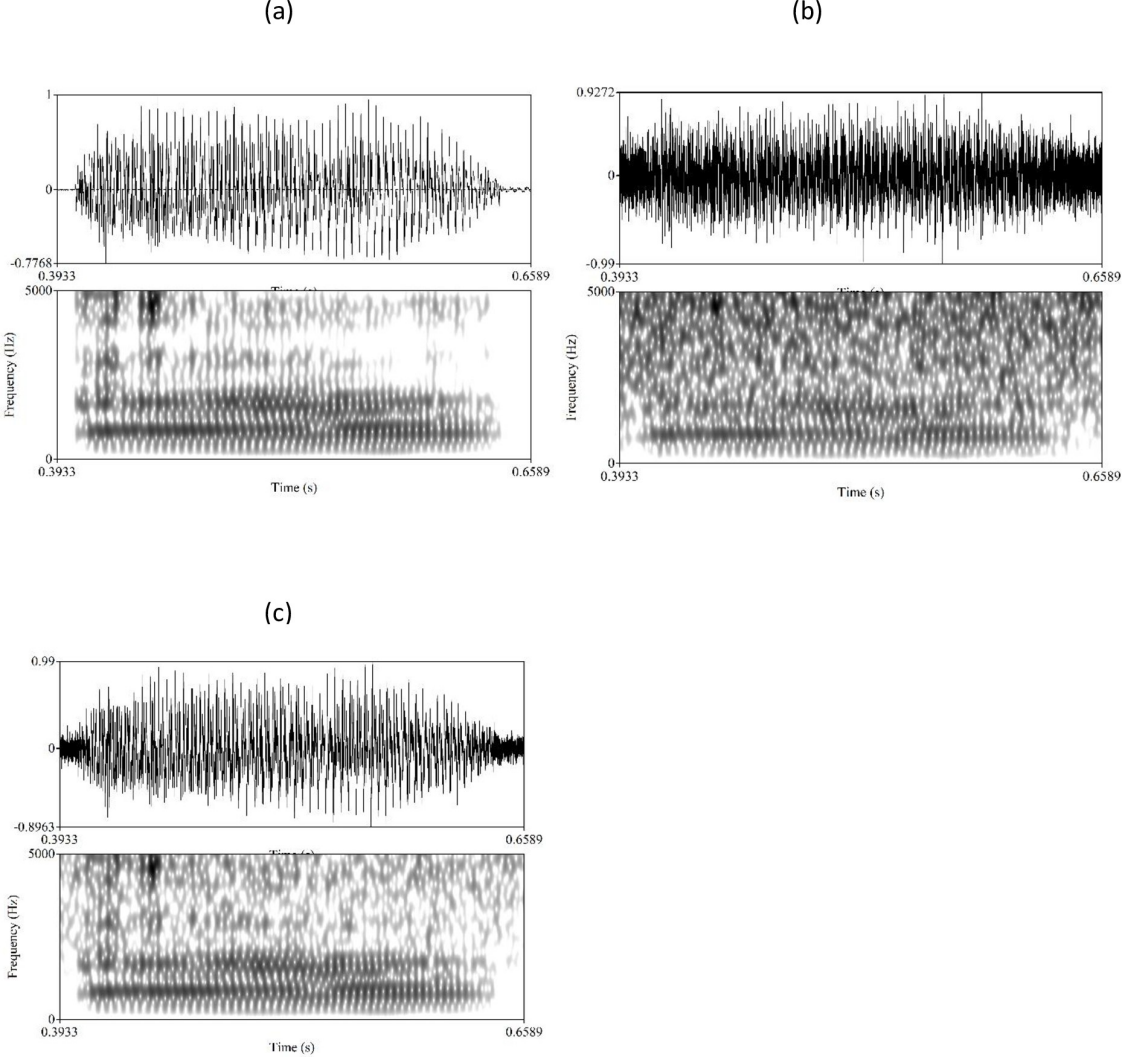
Waveforms and spectrograms of a speech stimulus. Original stimulus (a) and mixed with noise at SNR ratios of -6 dB (b) and +6 dB (c).

A subset of audiovisual stimuli were selected for experiment 2, and mixed with various levels of white noise (in line with [2]). For each vowel /i e y u/ produced by a given speaker, the fourth and sixth repetitions were selected. For each repetition, six versions were created using Praat by mixing the.wav file with noise in order to reach the following signal-to-noise ratio (SNR) values: –18 dB, –12 dB, –6 dB, 0 dB, 6 dB and 12 dB. Spectrograms of an original speech signal and one mixed with noise at various SNR ratios are shown in Fig 3. Thus, a total of 768 vowels (4 vowels x 2 repetitions x 6 SNR levels x 16 speakers) were obtained.

#### 2.2.2 Experimental procedure

Another group of 20 adult listeners (10 women, 10 men), aged between 21 and 35, were recruited for this second perceptual task. They were all native speakers of Canadian French living in the Montreal area. They did not report any history of speech or language disorders. All participants were tested for pure-tone detection threshold using an adaptive method (DT < 25 dB HL at 250, 500, 1000, 2000, 4000, and 8000 Hz). They all had perfect or near-perfect vision, or their vision was corrected using lenses or glasses. The 768 instances of vowels were presented audiovisually in random order using Presentation software (Version 18.0, Neurobehavioral Systems, Inc., Berkeley, CA). Listeners were wearing headphones. Their task was to identify, for each audiovisual stimulus, the vowel they had perceived among a forced choice of five responses: /i/, /e/, /y/, /u/, and “other.” The experiment took place in the recording room at UQAM’s Laboratoire de phonétique and lasted about 40 minutes. The experiment was approved by UQAM’s institutional review board.

### 2.3. Data analysis

#### 2.3.1 Identification scores across modalities

Confusion matrices were obtained from the total number of responses collected in each experiment, for each modality. In experiment 2, confusion matrices were produced for each SNR level. Percentage of correct identification scores were then computed. Linear mixed-effects models (LME) were built to test the significance of the different variables’ effects on the data, using the *lme4* package within R [43]. The dependent variable was the identification score and the fixed factors were group, vowel, and condition for experiment 1, and group, vowel, and SNR level for experiment 2. Participants were included in the model as random factors (slopes and intercepts). Post hoc analysis was performed with contrasts of least-square means using the *lsmeans* package [44]. The *p* values were corrected for multiple comparisons using the Tukey HSD method.

#### 2.3.2 Transmitted information scores

In order to assess the degree to which information conveyed by each vowel along the three phonetic features of height, place of articulation and rounding was perceived by the listeners, a sequential information analysis (SINFA), as developed by [45], was conducted. For a given feature-level matrix (see Table 1), this iterative algorithm successively partials out the contribution of feature categories to perception, with the end result providing the percentage of information correctly recovered by the perceivers for each individual feature considered. The version used for our analyses was implemented as a custom Matlab procedure, tested by correctly recomputing published values from [45,46]. A different LME model was computed with the data obtained from the SINFA for each experiment. The dependent variables were the transmitted information scores and the fixed effects were group, condition and phonetic feature for experiment 1, and group, SNR level and phonetic feature for experiment 2. Participants were included in the model as random factors (slopes and intercepts). Post hoc analysis was performed with contrasts of least-square means using the *lsmeans* package. The *p* values were corrected for multiple comparisons using the Tukey HSD method.

#### 2.3.3 Production-perception relationships

In order to assess the perceptual weight of visual and auditory cues produced by blind and sighted speakers, multiple regression analyses were performed. For the height feature, the percentage of transmitted information in experiment 2 was considered the dependent variable and the independent continuous variables were ACD value and lip height. The categorical factors were speaker group and SNR level. For place of articulation, the percentage of transmitted information was predicted from ACD value, speaker group and SNR level. Finally, for the rounding feature, speaker group, SNR level and lip width were included in the model as independent variables to predict the percentage of transmitted information. For all analyses, each variable’s effect size was assessed using partial eta-squared values (η_p_^2^).

## 3. Results

### 3.1 Experiment 1

**Fig 4:**
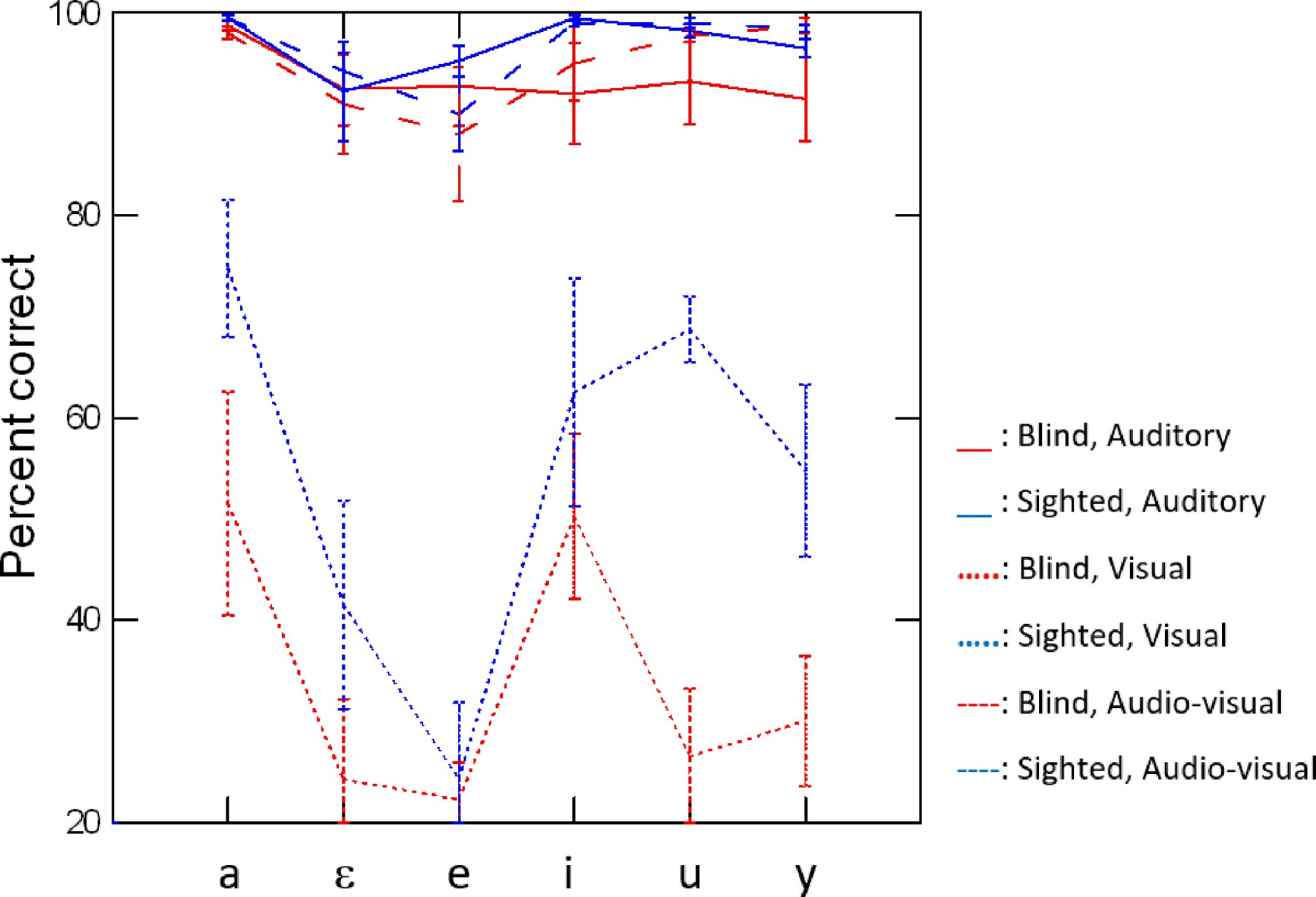
Percentages of correct responses averaged across listeners, for each vowel, in the auditory, visual, and audiovisual conditions.

Confusion matrices for the stimuli produced by the blind and sighted speakers are shown in Tables 3 and 4, for the three modalities. Intelligibility scores are displayed in Fig 4 for both speaker groups and all three conditions. First, as suggested by the responses in the visual condition, both vowels /u/ and /y/ correspond to one visual category (viseme), whereas vowels /i/, /e/, /ε/ and /a/ seem to form distinct visual categories. This featural structure will be further analyzed below. Furthermore, the pattern of results for /u/ and /y/ is asymmetrical across speaker groups. For blind rounded vowels presented in the visual condition, the produced /u/ was mostly perceived as /y/ (n = 278) and the produced /y/ was mostly perceived correctly (n = 299) with a substantial number of confusions with /u/ (n = 165). However, /u/ and /y/ produced by the sighted speakers yielded the reverse pattern in the visual condition: the produced /u/ was correctly identified in the majority of trials (n = 355) but the produced /y/ was mostly misidentified as /u/ (n = 277).

**Table 3:** Confusion matrices for vowels produced by the blind speakers in experiment 1.

	Produced ↓	Perceived					
		a	ε	e	i	u	y
**Audio**	a	475	3	0	0	0	0
	ε	4	450	25	0	0	1
	e	0	16	451	4	0	6
	i	0	0	13	448	0	19
	u	2	2	4	2	453	9
	y	0	3	14	16	0	446
**Audiovisual**	a	472	1	0	0	1	0
	ε	6	444	30	0	0	0
	e	2	19	432	24	0	2
	i	0	3	8	460	0	7
	u	0	0	0	0	471	4
	y	1	0	1	1	1	475
**Visual**	a	286	102	65	14	6	2
	ε	102	175	139	50	6	2
	e	30	127	169	119	7	4
	i	17	40	118	281	4	5
	u	1	0	0	0	186	278
	y	0	1	0	0	165	299

**Table 4:** Confusion matrices for vowels produced by the sighted speakers in experiment 1.

	Produced ↓	Perceived					
		a	ε	e	i	u	y
**Audio**	a	478	0	0	0	0	0
	ε	0	449	27	0	0	2
	e	0	1	461	13	0	2
	i	0	0	2	478	0	0
	u	2	0	0	0	473	3
	y	1	1	9	1	0	466
**Audiovisual**	a	478	0	1	0	0	0
	ε	0	457	19	0	0	0
	e	0	2	440	34	0	0
	i	0	0	1	476	0	1
	u	0	0	0	0	476	3
	y	0	0	0	0	0	474
**Visual**	a	379	78	16	2	1	5
	ε	73	246	106	50	1	6
	e	36	118	175	141	2	23
	i	21	34	93	320	2	14
	u	1	4	7	0	355	121
	y	1	3	9	1	277	200

The results of the LME model built on intelligibility scores suggest a significant main effect of condition (χ^2^(17) = 68.65; p<0.001), group (χ^2^(1) = 5.90; p<0.05) and vowel (χ^2^(20) = 53.26; p<0.001). Post hoc tests reveal that, overall, vowels produced by sighted speakers yielded higher percentages of correct responses than vowels produced by blind speakers (t(41.8) = –3.290; p<0.01). The percentages of correct responses in the visual condition are significantly lower than those in the auditory (t(18.4) = 19.99; p<0.001) and audiovisual (t(18.4) = 20.38; p<0.001) conditions. Importantly, a significant interaction between speaker group, vowel and condition was found (χ^2^(10) = 32.55; p<0.001). In the auditory and audiovisual conditions, none of the observed differences between speaker groups in Fig 4 reached significance. However, in the visual condition, intelligibility scores for /u/, /y/ and /a/ were significantly higher for stimuli produced by sighted speakers than for stimuli produced by blind speakers (/u/: t(118) = –6.53; p<0.001; /y/: t(118) = –5.40; p<0.01; /a/: t(118) = –5.12; p<0.01).

**Fig 5:**
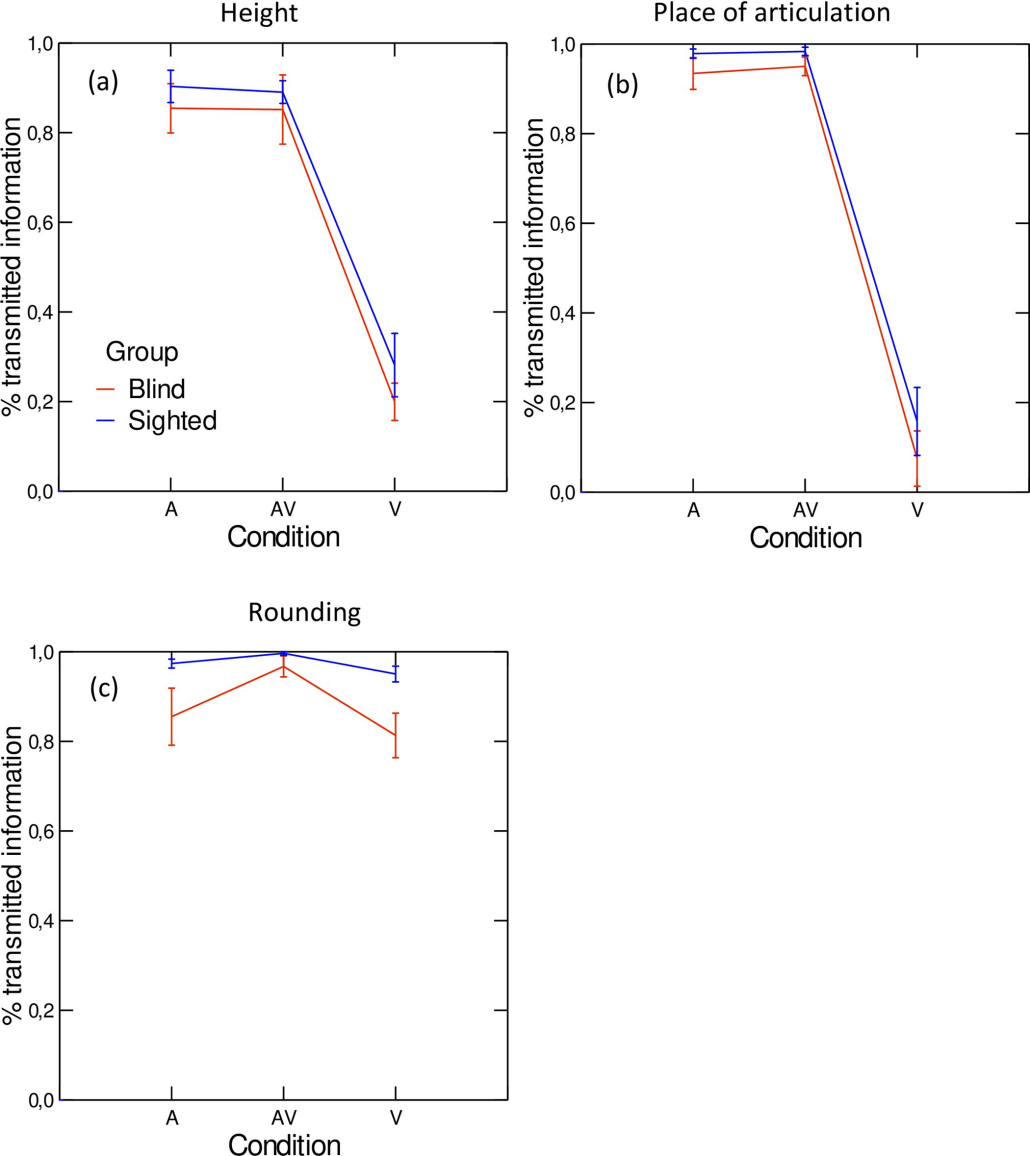
Percentages of transmitted information for each feature (height (a), place of articulation (b), and rounding (c)), in each condition, by speaker group. Error bars represent standard errors.

The results of the SINFA carried out on intelligibility scores are depicted in Fig 5, for the three features along which the perceived stimuli are contrasted: height, place of articulation, and rounding. The LME models revealed a significant main effect of speaker group (χ^2^(1) = 4.45; p<0.05), with sighted speakers producing vowels with an overall higher percentage of transmitted information than their blind peers. A significant main effect of condition was also found (χ^2^(2) = 136.95; p<0.001). Vowels presented in the auditory (A) and audiovisual (AV) conditions did not differ, but they were both associated with higher percentages of transmitted information than vowels presented in the visual (V) condition (A vs. V: t(90.2) = 23.94; p<0.001; AV vs. V: t(90.8) = 24.77; p<0.001)). Furthermore, the percentage of transmitted information significantly varies according to feature (χ^2^(2) = 47.87; p<0.001). Whereas place of articulation and height were transmitted at equal rates, those two features were both associated with lower transmission rates than the rounding feature (height vs. rounding: t(126) = –12.50); p<0.001; place of articulation vs. rounding: t(126) = –11.712; p<0.001). The rounding feature is also transmitted significantly worse in vowels produced by blind speakers than in vowels produced by sighted speakers (χ^2^(2) = 74.05; p<0.01). Finally, a significant two-way interaction of feature and condition was revealed by the LME (χ^2^(4) = 192.92; p<0.001): in the auditory and audiovisual modalities, all three features have similar percentages of transmitted information; in the visual modality, the height contrasts were significantly better transmitted than the rounding contrast (t(126) = –17.78; p<0.0001), which in turn was better transmitted than the place of articulation contrast (t(126) = –21.21; p<0.0001).

#### 3.2 Experiment 2

**Fig 6:**
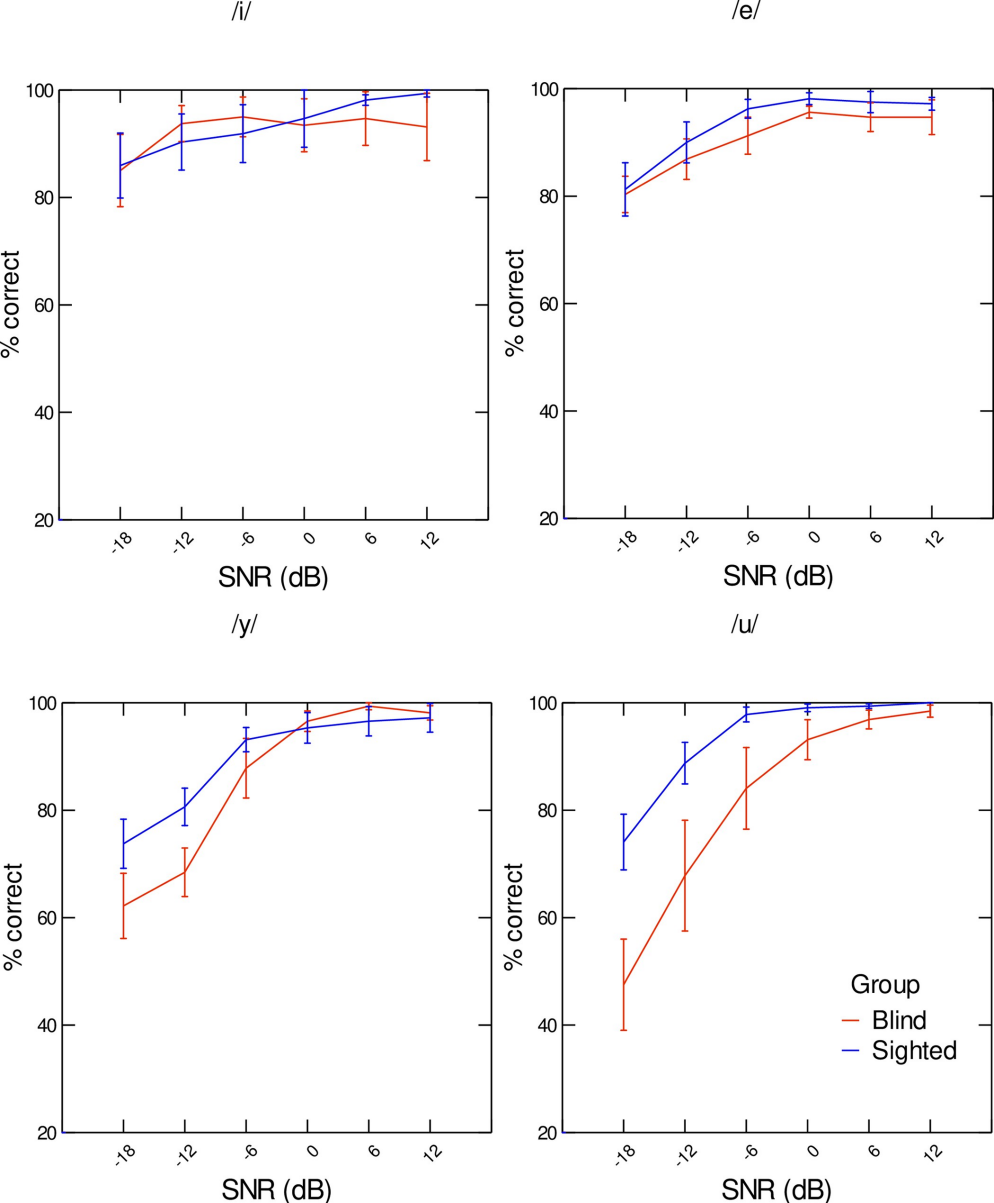
Percentages of correct responses (intelligibility scores) per SNR level, averaged across listeners, for the vowels /i/ (a), /e/ (b), /y/ (c), and /u/ (d).

The percentage of correct identification scores corresponding to each of the four vowels /i/, /e/, /y/, and /u/ that were used as stimuli in experiment 2 are presented in Fig 6, as a function of SNR level, for each speaker group. As the graphs show, overall, blind speakers produced vowels that yielded significantly lower percentages of correct responses than the vowels produced by the sighted participants (χ^2^(1) = 61.50; p<0.001). Moreover, as expected, SNR levels had a significant effect on intelligibility scores (χ^2^(5) = 178.86; p<0.001), as noisier conditions yielded lower percentage of correct identification scores: –18 dB vs. –12 dB (t(376) = –4.79; p<0.001) and –12 dB vs. –6 dB (t(376) = –4.42; p<0.001). Data also varied significantly according to vowel (χ^2^(3) = 20.02; p<0.001). The unrounded vowels /i/ and /e/ were associated with significantly higher correct identification scores than the rounded vowels /u/ and /y/ (t(376) = 2.904; p<0.01; t(376) = 3.50; p<0.001). Two-way interactions between speaker group and vowel (χ^2^(3) = 16.74; p<0.001), and between SNR level and vowel (χ^2^(15) = 67.69; p<0.001) also appeared. More importantly, the three-way interaction of SNR level, speaker group and vowel was found to be significant (χ^2^(15) = 42.59; p<0.01). Post hoc tests showed that percentage of correct identification scores were higher for vowels produced by sighted speakers than for vowels produced by blind speakers for the rounded vowels /y/ and /u/ at the lowest SNR levels: –18 dB SNR (/y/: t(313) = –5.63; p<0.001; /u/: t(313) = –4.84; p<0.01) and –12 dB SNR (/y/: t(313) = –5.02; p<0.01; /u/: t(313) = –4.79; p<0.01).

**Fig 7:**
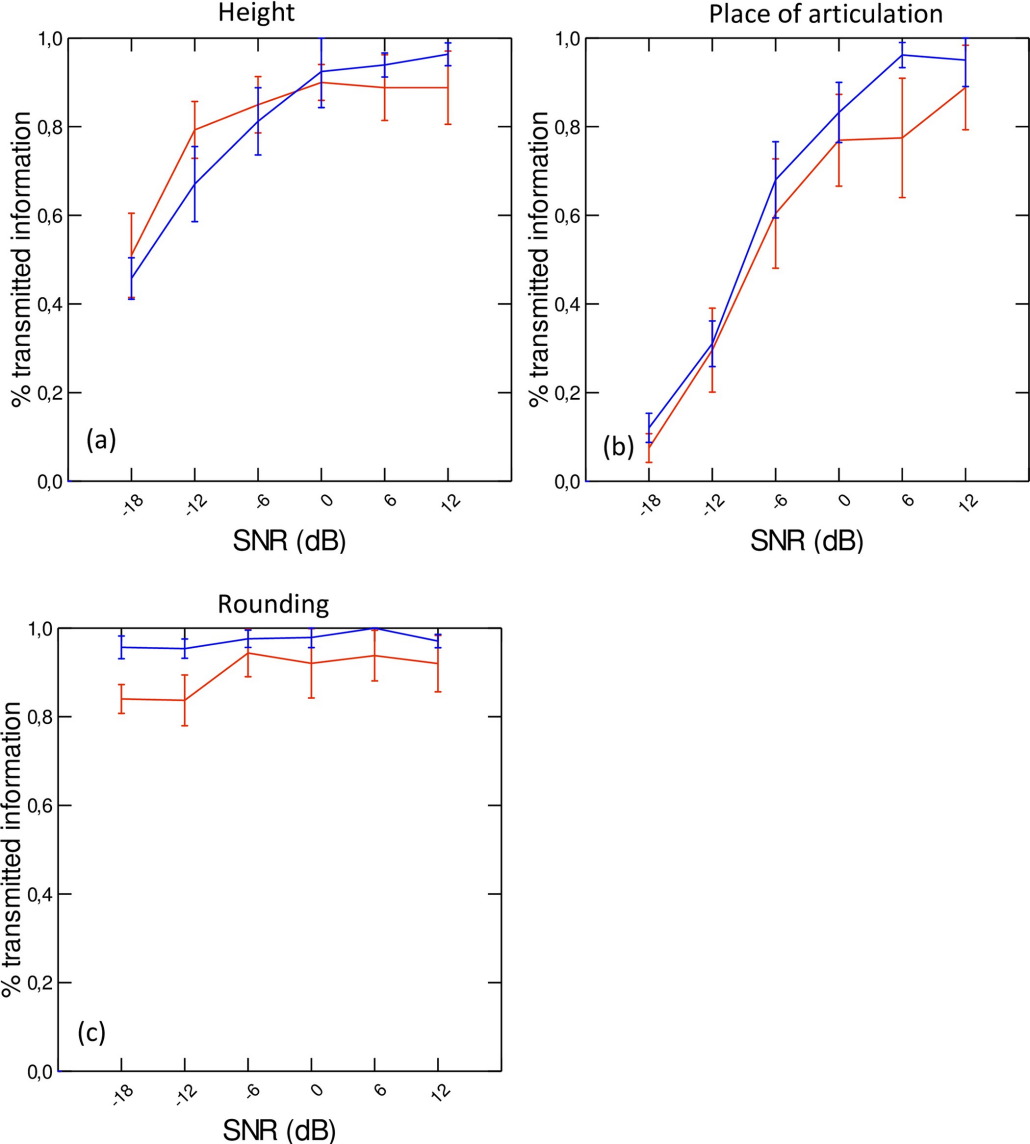
Percentages of transmitted information for each feature (height (a), place of articulation (b), and rounding (c)), by speaker group and SNR level. Error bars represent standard errors.

The results of the SINFA conducted on percentage of correct identification scores at the six SNR levels and for each speaker group are presented in Fig 7. As these figures show, the rounding feature is the one for which the percent information transmitted is the highest even in very noisy conditions (low SNR), whereas information related to place of articulation is almost lost in those conditions. The results of the LME model confirm the significant main effect of feature on the percentage of transmitted information (χ^2^(2) = 132.35; p<0.001): height is better transmitted than place of articulation (t(259) = 7.75; p<0.0001), which in turn is better transmitted than rounding (t(259) = –12.97; p<0.0001). When data are averaged across feature and speaker group, SNR levels also significantly affect the percentage of transmitted information (χ^2^(5) = 151.66; p<0.001). At SNR levels of –18 dB and –12 dB, significantly less information is recovered than at higher SNR levels (–18 dB: t(258) = –4.03; p<0.001; –12 dB: t(258) = –4.47; p<0.001). No significant main effect of group is found. The interaction between feature and condition is found to be significant (χ^2^(30) = 149.88; p<0.001). At an SNR level of –18 dB, rounding contrasts are better transmitted than height contrasts (t(192.4) = –8.47; p<0.0001), which in turn are better transmitted than place of articulation contrasts (t(192.4) = 7.88; p<0.0001). In less noisy conditions (–12 dB), only height is better transmitted than place (t(192.4) = 8.76; p<0.001). A significant three-way interaction between SNR level, feature and group is found (χ^2^(10) = 86.02; p<0.01): for blind speakers, compared to sighted speakers, in noisier conditions (SNR levels of –18 dB and –12 dB), the rounding feature is significantly less robust, whereas in quieter conditions (SNR levels of 6 dB and 12 dB), the place of articulation feature is significantly less robust.

### 3.3 Production-perception relationships

The results of the multiple regression analyses are presented in Table 5. F-values, p-values and partial eta-squared values (η_p_^2^) are listed for the factors that contributed significantly to the model. The regression models were all significant. For the height feature, the four factors SNR level, lip height, AVS and the interaction of lip height and AVS explained 70% of the variance in the percentage of transmitted information for that feature in sighted speakers and 69% for blind speakers. SNR level was the variable that had the largest effect on perception compared to other predictors for both speaker groups (η_p_^2^ = 0.67 for sighted speakers; η_p_^2^ = 0.61 for blind speakers). Data from Table 5 also show that ACD contributed more to the variance for vowels produced by the blind (η_p_^2^ = 0.30) than by the sighted speakers (η_p_^2^ = 0.15). Interestingly, lip height had a significant effect only for the perceived vowels produced by the sighted group (η_p_^2^ = 0.12). For the place of articulation feature, SNR level had a greater effect on the percentage of transmitted information for vowels produced by the sighted speakers (η_p_^2^ = 0.88) than for vowels produced by the blind speakers (η_p_^2^ = 0.64). As for rounding, ACD accounted for a significant proportion of the variance in transmitted information only for sighted speakers. For both groups, SNR level had a larger effect than lip width, although η_p_^2^ values were larger for sighted speakers than for blind speakers (SNR level: η_p_^2^ = 0.73 for sighted and η_p_^2^ = 0.35 for blind; lip width: η_p_^2^ = 0.37 for sighted and η_p_^2^ = 0.19 for blind).

**Table 5:** Results of multiple regression analyses. The dependent variable is the percentage of transmitted information for each feature.

	Predictor	F		p		η_p_^2^	
		Sighted	Blind	Sighted	Blind	Sighted	Blind
**Height**	**SNR**	12.38	16.36	<0.001	<0.001	0.61	0.67
	**Lip height**	5.67	ns	<0.05	ns	0.12	–
	**ACD**	6.80	17.45	<0.05	<0.001	0.15	0.30
**Place of articulation**	**SNR**	45.20	12.96	<0.001	<0.001	0.88	0.64
	**ACD**	0.96	3.45	ns	ns	–	–
**Rounding**	**SNR**	20.53	17.27	<0.001	<0.001	0.73	0.35
	**Lip width**	24.69	7.90	<0.001	<0.01	0.37	0.19
	**ACD**	7.01	1.75	<0.05	ns	0.15	–

## 4. Discussion

### 4.1. Review of hypotheses

The experiments described in this paper were designed to test the following hypotheses. First, based on our previous work on the acoustic characteristics of vowels produced by blind and sighted Canadian French speakers, we hypothesized that, when presented in the auditory modality, vowels produced by sighted speakers would be associated with higher intelligibility scores than vowels produced by blind speakers. Although overall, when we average across conditions and vowels, vowels produced by blind speakers are less intelligible than vowels produced by sighted speakers (experiment 1), this hypothesis was not confirmed for the auditory modality (Fig 4A). In this modality, vowels produced by the two speaker groups did not differ significantly in terms of intelligibility. In the visual modality, we hypothesized that vowels produced by blind speakers would be associated with lower scores than vowels produced by sighted speakers. This hypothesis was confirmed. As shown in Fig 4, the three vowels /a/, /u/ and /y/ had significantly lower percentages of correct responses when they were produced by blind speakers than when they were produced by sighted speakers. This pattern is, however, feature-dependent: the rounding feature is significantly less well identified in vowels produced by the blind group than in vowels produced by the sighted group (Fig 5C).

**Fig 8:**
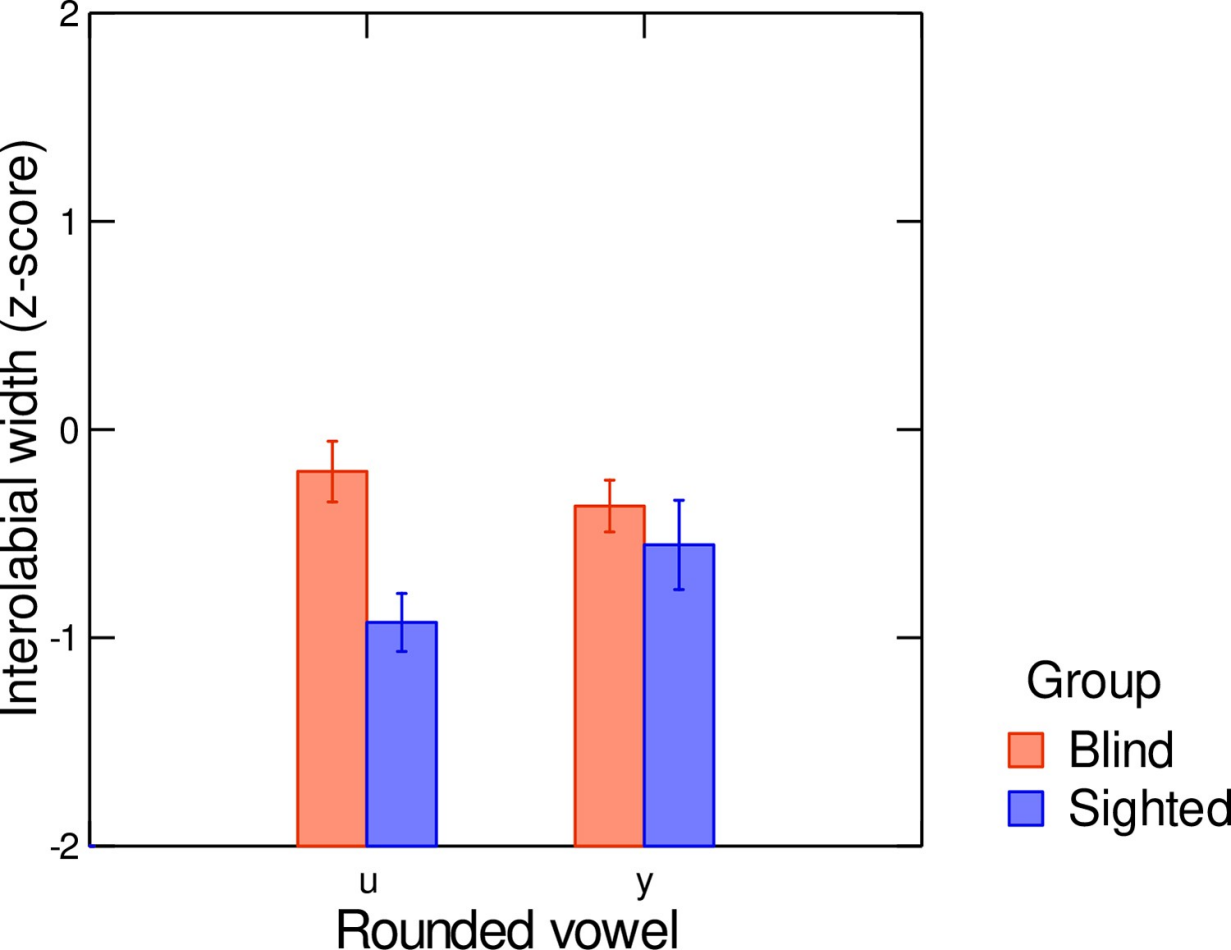
Average values of interolaboal width of vowel stimuli /u/ and /y/ produced by blind and sighted speakers. All data were z-scored. Error bars are standard errors.

Finally, hypothesis 3 stated that, in the audiovisual condition, intelligibility scores would be lower for blind than for sighted speakers. This hypothesis was partly confirmed. Indeed, as shown by the results of experiment 1, in non-noisy conditions, vowels produced by blind and sighted speakers had similar intelligibility scores. This result suggests that, despite the reduced magnitude of lip movements associated with vowels produced by blind speakers compared to sighted speakers (see Fig 2), the acoustic cues provided by the blind speakers compensate for the lack of visual saliency of their lip gestures. However, in noisy conditions, a different pattern is observed (see Figs 6 and 7). At lower SNR levels (–18 dB and –12 dB), lower identification scores are associated with the rounded vowels /u/ and /y/ produced by the blind speakers compared to the sighted speakers. This tendency is found for /u/ even in less noisy conditions (SNR levels of –6 dB and 0 dB). To better understand this pattern of results, we have extracted the average interolabial width associated with the stimuli /u/ and /y/ produced by both speaker groups (see Fig 8). As can be seen in Fig 8, while lip width is smaller in /u/ than in /y/ for sighted speakers (as reported in [2]), the reverse pattern (although to a lesser extent) is seen in blind speakers. The group difference is particularly salient for /u/, for which blind speakers produced larger lip width than sighted speakers. This is in line with our previous findings that place of articulation and rounding are implemented by different lip and tongue settings in sighted and blind adults (for instance [11,12]). It is not surprising, then, that /u/ produced by blind speakers was mostly misidentified as /y/. This result might be related to the fact that the first two formants of /u/ are affiliated with two cascade Helmholtz resonators [47,48]. In such configurations, resonant frequencies depend on a combination of several parameters: cavity volume, constriction area and constriction length (unlike tube resonances). As a consequence, speakers have to precisely control lip and tongue configurations to produce appropriate Helmholtz resonators corresponding to low F1 and F2. In sighted speakers, it is likely that the perceived visual cues provided by the lips during speech development constrain the produced lip configurations associated with /u/ early on, before tongue control reaches maturity. No such constraining cues are available to blind speakers, resulting in alternative lip-tongue configurations for producing this vowel.

At the featural level, the SINFA confirms that rounding is less robust for blind than for sighted speakers at lower SNR levels. Interestingly, at higher SNR levels (+6 dB and + 12 dB), the place of articulation feature is transmitted less well by blind speakers than by sighted speakers. Thus, in noisy audiovisual conditions, when perceivers rely primarily on visual cues to identify phonemic information, vowels produced by blind speakers, who present reduced contrasts between lip positions compared to sighted speakers, are associated with less rounding identification. In quieter conditions, in which auditory cues are weighted more to identify vowels, intelligibility scores are still lower for blind speakers than for sighted speakers in terms of place of articulation. Thus, the reduced acoustic vowel space typical of blind francophone speakers does not totally compensate for the low visual saliency of the orofacial articulators, in multimodal conditions.

### 4.2. Production-perception relationships

To further investigate production-perception relationships, we computed mixed regression analyses. Based on the effect sizes (η_p_^2^), different patterns were observed for the two speaker groups and the three vowel features. For vowels contrasting along the height dimension, the visually salient information related to lip height did not significantly contribute to the perception of this feature, as produced by blind speakers, contrary to sighted speakers. For the place of articulation feature, which has been reported to be primarily transmitted through the acoustic-auditory channel, the larger effect size of SNR levels on place identification in sighted speakers (η_p_^2^ = 0.88) than in blind speakers (η_p_^2^ = 0.64) confirms that, in less noisy conditions (higher SNR levels), acoustic-auditory cues are more salient in the vowels produced by the former group than by the latter. A similar pattern can be found for the rounding feature. Although SNR level and lip width significantly predicted the perceived variance for both sighted and blind speakers’ vowels, both variables had much larger effect sizes for the former than for the latter. Again, this result confirms that visually relevant perceptual cues are more reliably found in vowels produced by sighted speakers than in those produced by blind speakers. Similarly, auditory perceptual cues (found in ACD values as SNR level increases) are less strongly conveyed by vowels uttered by blind speakers than by sighted speakers. This study confirms that, despite the trade-off between tongue and lip displacements found in vowel production by blind and sighted francophone adults, the multimodal intelligibility of the resulting vowels produced by blind speakers do not reach the levels found for sighted speakers. Thus, the greater magnitude of tongue movements –an invisible articulatory movement –does not totally compensate for the reduced visible lip movement contrasts. Further studies focusing on the developmental path of speech production and perception in blind individuals are currently under way.

### 4.3. Limitations of the current study

Although the subset of vowels that we selected as stimuli have similar acoustic characteristics to those calculated for blind individuals in larger data sets, as described in section 2.1.1 (Fig 2B), they do not cover the entire French vowel space. In particular, back mid-high and mid-low vowels should be investigated to ensure that the results can be generalized to the whole vowel space. Furthermore, as the standard errors depicted in Figs 3 to 5 reveal, in some cases larger between-listener variability is found for stimuli produced by blind individuals than for those produced by sighted individuals. Whether this variability is due to listener responses or to variability among the blind speakers has yet to be investigated. Some blind speakers might produce vowels for which the visual and acoustic cues are quite similar to those produced by the sighted speakers and, as such, may be associated with higher perceptual scores. Such variability has also been reported in our previous studies of blind speech. Finally, an issue that needs further consideration is the possibility of overestimating the percentage of transmitted information by applying SINFA to relatively small data sets; however, the effects of small-sample bias on SINFA are not currently known [49]. However, as Sagi & Svirsky (2008) observe [49], while the existence of such bias cannot be ruled out, in a study like ours, where the primary goal is to test the relative effects of speaker group (or any other variable) on percent transmitted information rather than to quantify transmission in absolute terms, SINFA can nonetheless be considered an appropriate method of analysis.
